# Lysophosphatidic Acid Receptor 1 Specifically Labels Seizure-Induced Hippocampal Reactive Neural Stem Cells and Regulates Their Division

**DOI:** 10.3389/fnins.2020.00811

**Published:** 2020-08-14

**Authors:** Roberto Valcárcel-Martín, Soraya Martín-Suárez, Teresa Muro-García, Oier Pastor-Alonso, Fernando Rodríguez de Fonseca, Guillermo Estivill-Torrús, Juan Manuel Encinas

**Affiliations:** ^1^The Neural Stem Cell and Neurogenesis Laboratory, Achucarro Basque Center for Neuroscience, Leioa, Spain; ^2^Department of Neurosciences, University of the Basque Country (UPV/EHU), Leioa, Spain; ^3^Unidad de Gestión Clínica de Salud Mental, Hospital Regional Universitario de Málaga, Instituto de Investigación Biomédica de Málaga (IBIMA), Málaga, Spain; ^4^Unidad de Gestión Clínica de Neurociencias, Hospital Regional Universitario de Málaga, Instituto de Investigación Biomédica de Málaga (IBIMA), Málaga, Spain; ^5^Ikerbasque, The Basque Foundation for Science, Bilbao, Spain

**Keywords:** neural stem cells, hippocampal neurogenesis, seizures, lysophosphatidic acid receptor 1, gliosis

## Abstract

A population of neural stem cells (NSCs) dwelling in the dentate gyrus (DG) is able to generate neurons throughout adult life in the hippocampus of most mammals. These NSCs generate also astrocytes naturally and are capable of generating oligodendrocytes after gene manipulation. It has been more recently shown that adult hippocampal NSCs after epileptic seizures as well as subventricular zone NSCs after stroke can give rise to reactive astrocytes (RAs). In the hippocampus, the induction of seizures triggers the conversion of NSCs into reactive NSCs (React-NSCs) characterized by a drastic morphological transformation, abnormal migration, and massive activation or entry into the cell cycle to generate more React-NSCs that ultimately differentiate into RAs. In the search for tools to investigate the properties of React-NSCs, we have explored the LPA_1_–green fluorescent protein (GFP) transgenic line of mice in which hippocampal NSCs are specifically labeled due to the expression of lysophosphatidic acid receptor 1 (LPA_1_). We first addressed the validity of the transgene expression as true marker of LPA_1_ expression and then demonstrated how, after seizures, LPA_1_-GFP labeled exclusively React-NSCs for several weeks. Then React-NSCs lost LPA_1_-GFP expression as neurons of the granule cell layer started to express it. Finally, we used knockout for LPA_1_ transgenic mice to show that LPA_1_ plays a functional role in the activation of React-NSCs. Thus, we confirmed that LPA_1_-GFP expression is a valid tool to study both NSCs and React-NSCs and that the LPA_1_ pathway could be a target in the intent to preserve NSCs after seizures.

## Introduction

New neurons are generated postnatally and into adulthood in the dentate gyrus (DG) of the hippocampus of the majority of mammals (Altman and Das, 1965; Bonfanti and Peretto, 2011). Adult hippocampal neurogenesis (AHN) is maintained, thanks to a population of astrocyte-like and radial glia-like neural stem cells (NSCs) with neurogenic (Seri et al., 2001) and gliogenic potential (Encinas et al., 2011). The population of hippocampal NSCs dwells specifically in the subgranular zone (SGZ) and generates neurons that get integrated into the granule cell layer (GCL). Adult hippocampal neurogenesis has been described to be involved in spatial learning (Zhang et al., 2008), memory (Farioli-Vecchioli et al., 2008), and forgetting (Akers et al., 2014). Moreover, the lack of AHN associates with increased anxiety and altered the responses to stress and fear (Saxe et al., 2006; Bergami et al., 2008; Snyder et al., 2011). Finally, antidepressant drugs present neurogenesis-dependent effects (Sharma et al., 2007; David et al., 2009; Perera et al., 2011). The properties of NSCs determine the neurogenic and gliogenic output of the DG in normal and pathological conditions. Hippocampal NSCs are mostly quiescent, and only a small fraction (around 2%) is actively cycling in a given moment (Kronenberg et al., 2003; Encinas et al., 2006). Because gliogenic or neurogenic differentiation following entry into the cell cycle is the main factor driving the depletion of the NSC pool (Bonaguidi et al., 2011; Encinas et al., 2011; Pilz et al., 2018), regulation of the quiescence/activation equilibrium is key to sustained neurogenesis throughout adulthood as self-renewing symmetric division does not compensate exhaustion *in vivo* (Bonaguidi et al., 2011; Pilz et al., 2018). Neuronal activity tightly regulates NSC activation through excitatory and inhibitory neuronal input to NSCs (Song et al., 2012; Yeh et al., 2018).

We recently showed how in mouse models of mesial temporal lobe epilepsy (MTLE) hippocampal seizures induced by intrahippocampal (Sierra et al., 2015) or intra-amygdalar (Muro-García et al., 2019) injection of the glutamate receptor agonist kainic acid (KA) strongly affect NSCs. Seizures trigger a dramatic switch in the phenotype and function of NSCs. Shortly after the initial episode of seizures, NSCs convert into reactive NSCs (React-NSCs) that are characterized by several features: (1) they transform into a more complex multibranched shape and overexpress the neuroectodermal stem cell marker (nestin) and the glial fibrillary acidic protein (GFAP); (2) they detach from the SGZ and migrate into the GCL; (3) they get activated in large numbers switching to a symmetric mode of division to generate more React-NSCs; and (4) they finally transform, after 4–6 weeks, into S100β-positive reactive astrocytes (RAs) morphologically indistinguishable from astrocyte-derived RAs. Thus, React-NSCs represent a novel pathophysiological cell fate of NSCs that we aim to investigate.

One of the major problems to study the properties of NSCs and, in our case, React-NSCs is the lack of pure specific biomarkers. Traditional markers such as GFAP, sex determining region Y-box 2 (Sox2), vimentin, brain lipid-binding protein, and others are also expressed by astrocytes and RAs, thus making biomarker-based identification of NSCs and React-NSCs difficult. Recently, the lysophosphatidic acid receptor 1 (LPA_1_) has been reported as a new specific marker of adult NSCs. A transgenic mouse line in which the enhanced green fluorescent protein expression (GFP) driven by LPA_1_ (LPA_1_-GFP) expression labels hippocampal NSCs has been described (Walker et al., 2016). We herein investigated whether LPA_1_-GFP mice could be a useful tool to identify and investigate React-NSCs. Further, LPA_1_ has been linked to regulation of neurogenic process. The brain of a mouse strain lacking LPA_1_, the *Málaga* variant of LPA_1_-null (maLPA_1_-null), has shown defects in cortical layer width and reduced proliferation and neurogenesis in the adult DG (Estivill-Torrus et al., 2008; Matas-Rico et al., 2008). Also, the endogenous ligand LPA induced AHN *in vivo* (Walker et al., 2016). We have here used the maLPA_1_ mice in our model of MTLE to investigate whether it plays a role in the seizure-induced transformation of NSCs into React-NSCs.

## Materials and Methods

For an extended description of methods, see section “Materials and Methods” and “Supplementary Materials and Methods” in Supplementary Material.

### Animals

Lysophosphatidic acid receptor 1–GFP transgenic mice, generated by the GENSAT project at Howard Hughes Medical Institute (The Rockefeller University, NY, United States) (Gong et al., 2003), were provided by Dr. Gerd Kempermann at the Center for Regenerative Therapies Dresden (Technische Universität Dresden, Dresden, Germany) and crossbred with C57BL/6 mice for at least 10 generations. maLPA_1_-null mice (Estivill-Torrus et al., 2008), derived from previously reported LPA_1_-null mice (Contos et al., 2000), and their wild-type (WT) counterparts (on a mixed background C57BL/6 × 129SW) were kindly provided by Guillermo Estivill-Torrús at the Instituto de Investigación Biomédica de Málaga (IBIMA, Hospital Regional Universitario de Málaga, Málaga, Spain). All procedures were approved by the University of the Basque Country (UPV/EHU) Ethics Committees (Leioa, Spain) and Diputación Foral de Bizkaia under protocol M20/2015/236. All procedures followed the European directive 2010/63/UE and National Institutes of Health guidelines.

### 5-Bromo-2′-Deoxyuridine Administration

5-Bromo-2′-deoxyuridine (BrdU) was administered intraperitoneally (four injections 2 h apart) on the second day after the intrahippocampal injection.

### Immunohistochemistry and Cell Quantification

Immunostaining of brain slices, image capture, and quantitative analysis were performed essentially as described before following methods optimized for their use in transgenic mice (Encinas et al., 2011; Sierra et al., 2015; Muro-García et al., 2019).

### Model of MTLE (The Stereotaxic Intrahippocampal Injection of KA)

We followed the protocol optimized and described in Sierra et al. (2015), Abiega et al. (2016), and Bielefeld et al. (2017). We used the coordinates anteroposterior 1.8 mm, laterolateral 1.6 mm, and dorsoventral 1.9 mm taken from Bregma (Franklin and Paxinos, 1997). Controls were injected with 50 nL of saline and MTLE mice with 20 mM KA (1 nmol) dissolved in saline.

### Statistical Analysis

For analyses involving more than two groups, a one-way analysis of variance (ANOVA) was performed (postnatal and neuronal expression of LPA_1_-GFP). When evaluating the time × treatment interaction in the LPA_1_-GFP time course, a two-way ANOVA was employed, and analyses continued when normality assumptions were fulfilled. When comparing two groups (integrated density) a Mann-Whitney U test was used. Otherwise data were transformed into logarithmic scale. *Post hoc* Holm–Sidak test followed in all cases.

## Results

**FIGURE 1:**
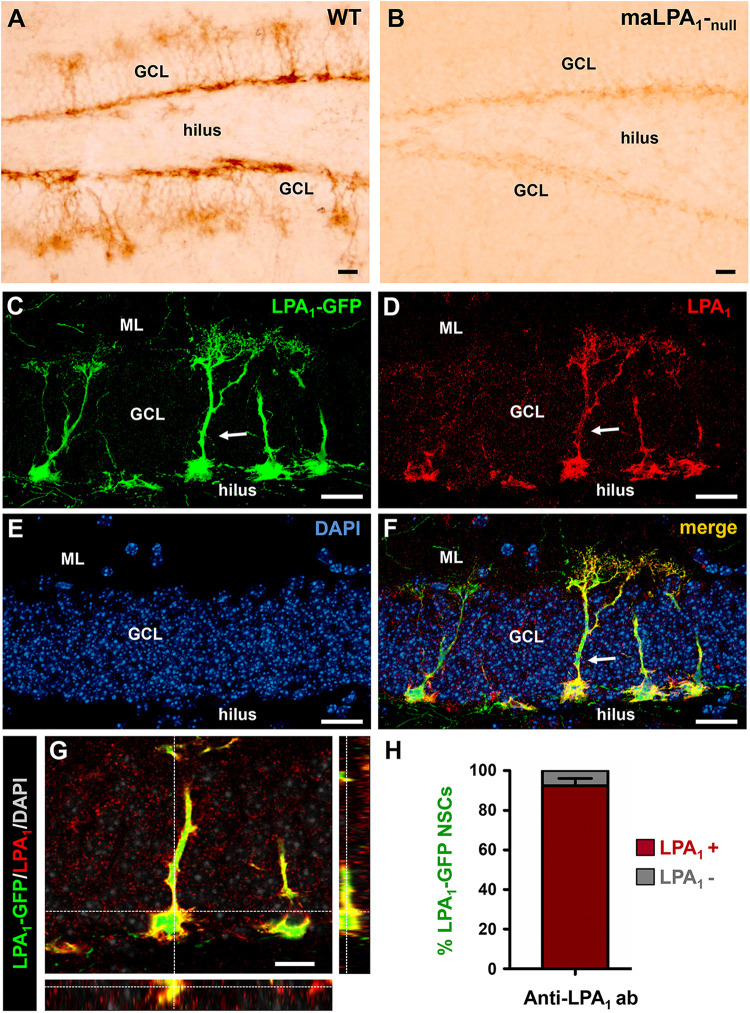
Lysophosphatidic acid receptor 1–GFP expression colocalizes with LPA_1_ immunostaining and specifically labels hippocampal NSCs. Immunostaining with an anti-LPA_1_ antibody labels NSCs in the DG **(A)**. When the same antibody is used in the maLPA_1_-null mouse, in which only a truncated non-functional form of LPA_1_ is expressed, staining is almost absent **(B)**. Immunostaining with the anti-LPA_1_ antibody in slices from the LPA_1_-GFP transgenic mouse shows almost total colocalization. Confocal microscopy imaging was used to analyze colocalization in z-stack projections of the LPA_1_-GFP signal **(C)** with the anti-LPA_1_ immunostaining **(D)**. DAPI staining was used for better anatomical resolution **(E)**. The merged image is shown in **(F)**, and an orthogonal projection demonstrating full colocalization is shown in **(G)**. The quantification showing the almost 100% colocalization of LPA1-GFP and LPA1 immunostaining in NSCs is shown in **(H)**. Scale bar is 20 μm in all images.

A previous description of the LPA_1_-GFP transgenic line of mice showed the specificity of LPA_1_-GFP expression for marking the NSC population of the adult hippocampus (Walker et al., 2016). In order to validate the *bona fide* expression of GFP in LPA_1_-expressing cells, we used immunostaining with an anti-LPA_1_ antibody. We first used the anti-LPA_1_ antibody in tissue from WT mice and from *Málaga* LPA_1_-null mice, (maLPA_1_-null mice), previously characterized (Estivill-Torrus et al., 2008). Staining in the WT mice labeled NSCs in the hippocampus, whereas staining was almost absent in the maLAP_1_-null sections (Figures 1A,B). Neural stem cells were identified because of their unique morphology and location. Their soma is located along the SGZ between the hilus and the GCL, and a single apical process extends though the GCL toward the molecular layer. There it branches profusely in a broccoli-like arborization. Light staining was found in the maLPA_1_-null mice because a residual level of a truncated form of LPA_1_ is expressed (Figure 1B). We next validated the specificity of the expression of GFP by assessing the localization of LPA_1_ immunostaining in brain sections from LPA_1_-GFP mice. We confirmed that the vast majority of LPA_1_-GFP^+^ NSCs expressed LPA_1_, thus confirming the reliable correspondence between LPA_1_-GFP and endogenous LPA_1_ expression (Figures 1C–H) in normal conditions. Importantly, no other cell type expressed LPA_1_-GFP in the hippocampus. These results are in agreement with an aging-related study in which we tested the expression of the LPA_1_ protein in 8 month-old nestin-GFP mice and showed that more than 90% of nestin-GFP^+^/GFAP^+^ NSCs costained with the anti-LPA_1_ antibody (Martín-Suárez et al., 2019). Noteworthy, immunohistochemistry for the LPA_1_ required a specific fixation protocol (see *Materials and Methods*) that was not compatible with staining for most of the antibodies used in this study. This issue underscores the usefulness of the LPA_1_-GFP transgenic line of mice. We next analyzed the expression of LPA_1_-GFP in early postnatal and young mice and found that LPA_1_ is absent from radial nestin-expressing NSCs located in SGZ and GCL in newborn mice, starting in postnatal day 4 (P4), but its expression increased gradually over time until it was present in the vast majority of them by P14 (Supplementary Figures 1A,B). Interestingly, an abundant population of LPA_1_-GFP–expressing neurons (colocalizing with the neuronal marker NeuN) was found in P4 in the hilus (Supplementary Figure 1C). In an opposite fashion to NSCs, hilar neurons lost LPA_1_-GFP expression over time, and it was almost absent by P14.

**FIGURE 2:**
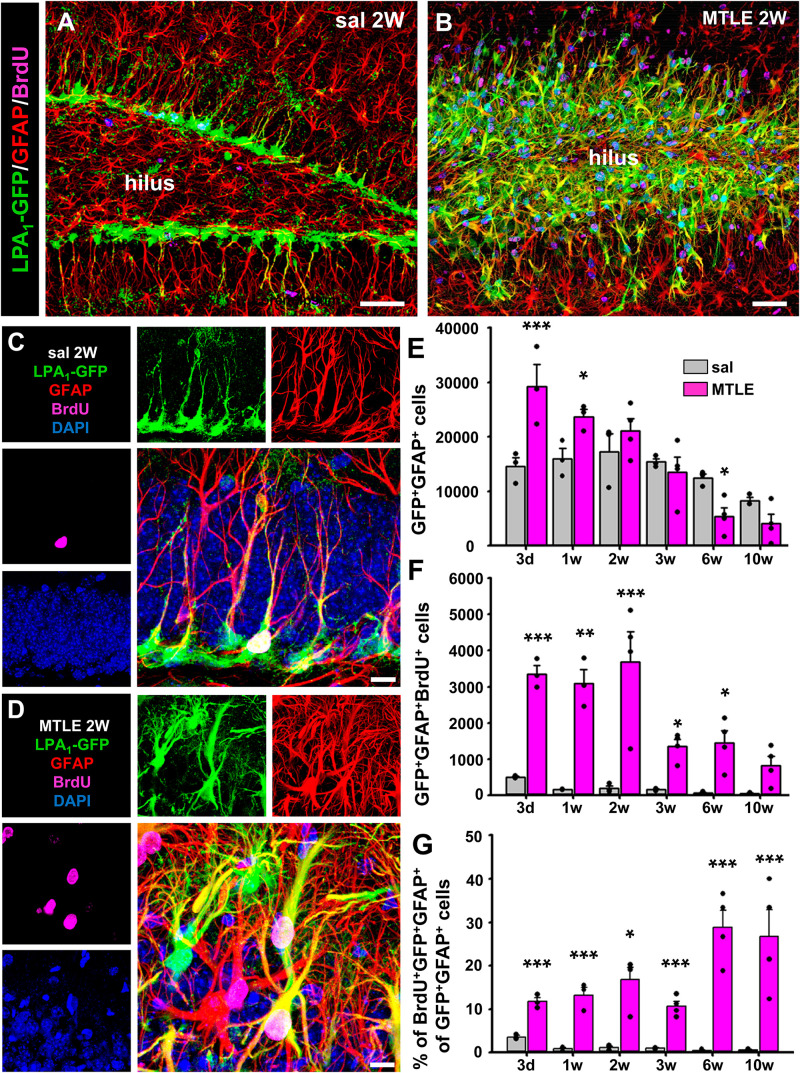
Lysophosphatidic acid receptor 1–GFP expression is maintained in seizure-induced React-NSCs. Control and MTLE mice were administered BrdU (four injections, 2 h apart) on the second day after surgery for the saline/KA intrahippocampal injection and then sacrificed 3 days or 1, 2, 3, 6, or 10 weeks after surgery. Expression of LPA_1_-GFP is restricted to GFAP-expressing NSCs in the SGZ in the control mice **(A)** and to GFAP-expressing React-NSCs located in the SGZ or GCL in the MTLE mice **(B)**. In the molecular layer, GFAP-expressing astrocytes did not express LPA_1_-GFP as shown by z-stack projections obtained by confocal microscopy. The time-course analysis with BrdU costaining at higher magnification **(C,D)** shows an overall increase in the number of LPA_1_-GFP^+^GFAP^+^ cells in early time points and a later decline **(E)**. The number of BrdU^+^ LPA_1_-GFP^+^GFAP^+^ cells is much higher in MTLE mice in every time point, but it decreases in the later ones **(F)**. The percentage of BrdU^+^ LPA_1_-GFP^+^GFAP^+^ follows an opposite trend as it increases over time, although it is also much higher than in controls in all time points **(G)**. Scale bar is 50 μm in **(A,B)** and 10 μm in **(C,D)**. Data were analyzed by a two-way ANOVA. *Post hoc* Holm–Sidak was used to analyze differences between time points and sal vs. MTLE. Only the differences between sal vs. MTLE in each time are shown. *p < 0.05, **p ≤ 0.005, ***p ≤ 0.001.

Our main hypothesis is that seizure-induced React-NSCs could keep expressing LPA_1_ that could be therefore a useful specific marker of this cell type. Thus, we designed a time-course experiment in which LPA_1_-GFP mice were subjected to the hippocampal model of MTLE (a single intrahippocampal injection of KA). On the second day after they were given four intraperitoneal injections of 150 mg/kg of BrdU (2 h apart). Mice were then sacrificed on the third day or after 1, 2, 3, 6, or 10 weeks. As reported before, the presence of normal NSCs with typical radial morphology was negligible in the DG of the MTLE mice (Figures 2A,B). In the MTLE mice, LPA_1_-GFP cells with reactive morphology (Figures 2B,D) were located mainly in the SGZ and the GCL. Granule cell layer dispersion with a subsequent narrowing of the hilus is a hallmark of human and experimental MTLE. As a result, LPA_1_-eGFP–expressing cells occupy a broader area of the DG in MTLE mice than in control ones. Noteworthy, LPA_1_-GFP expression was absent in the molecular layer, in which RAs are induced by MTLE. We observed that React-NSCs kept expressing LPA_1_-GFP but that no other cell type did in the earlier time points (Figures 2A–C) as LPA_1_-GFP expression was restricted to GFAP^+^ cells located in the SGZ and GCL. The overall number of LPA_1_-GFP^+^GFAP^+^ cells in MTLE mice was significantly higher than in control mice during the time course, especially at the earliest time points. It, however, decreased over time being even lower than controls by 6 weeks (Figure 2E). In agreement with our previous reports, React NSCs, labeled with LPA_1_-GFP^+^, entered cell division with a much higher rate than control NSCs from intrahippocampally saline-injected mice (Figures 2C,D). The total number of LPA_1_-GFP^+^GFAP^+^BrdU^+^ cells was significantly much higher in MTLE than in saline mice, although it decreased overtime and was close to controls after 10 weeks (Figures 2C,D,F). The proportion of BrdU^+^/LPA_1_-GFP^+^/GFAP^+^ among the total LPA_1_-GFP^+^/GFAP^+^ was significantly much higher in MTLE mice than in controls, and interestingly, it increased over time (Figure 2G).

**FIGURE 3:**
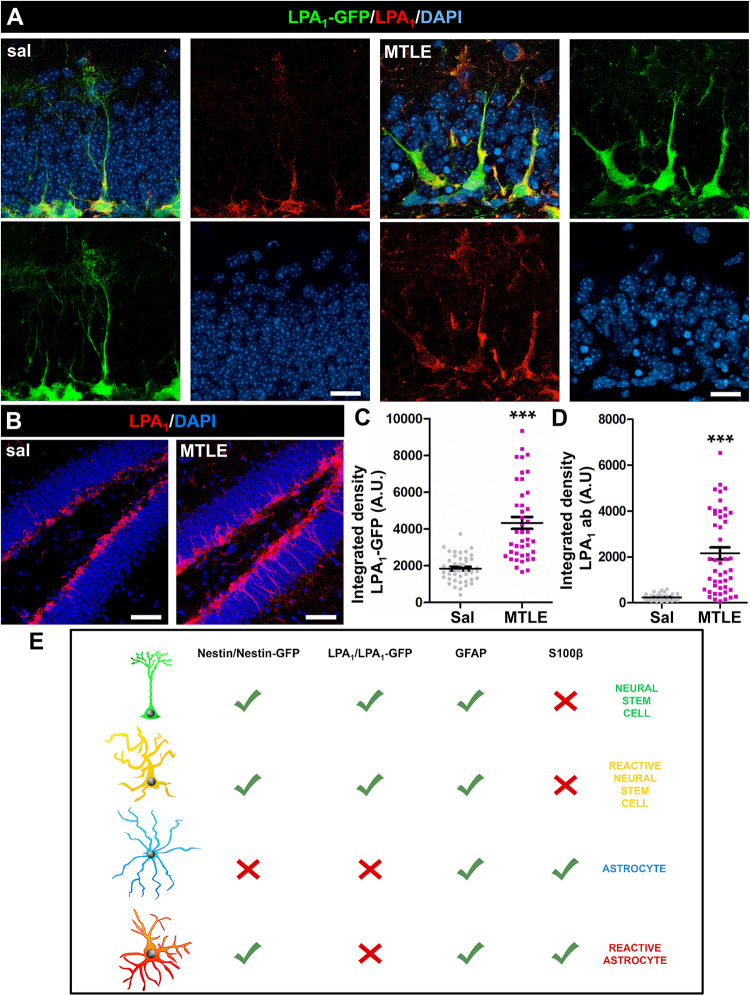
Neural stem cells in sal-injected (**A**, left) and React-NSCs in MTLE LPA_1_-GFP mice (**B**, right) express LPA_1_ (3 days after surgery) as demonstrated by immunostaining with anti-LPA_1_ antibody. No other cell types were labeled with either LPA_1_-GFP or anti-LPA_1_ antibody in control or MTLE mice **(B)**. The expression of LPA_1_-GFP **(C)** or LPA_1_ **(D)** was significantly increased in MTLE-induced React-NSCs compared to control NSCs as measured by the integrated density of the fluorescent signal. **(E)** shows the combination of biomarker expression that can be used to differentially identify NSCs, React-NSCs, astrocytes, and RAs. Scale bar is 10 μm in **(A)** and 50 in μm **(B)**. Data were analyzed by a Mann-Whitney U test. Graphs show individual data and mean ± SEM. ***p ≤ 0.001.

Importantly, we compared the expression of LPA_1_ by immunostaining with that of LPA_1_-GFP in the MTLE model (a single injection of KA into the hippocampus and analysis 3 days later). Lysophosphatidic acid receptor 1–GFP^+^ NSCs in the control mice and MTLE-induced React-NSCs were immunostained for LPA_1_ (Figures 2B, 3A,B). The labeling was restricted to the SGZ and the GCL and was absent in the rest of the areas of the DG, suggesting that even in the MTLE mice no other cell type such as astrocytes or RAs expressed LPA_1_. In addition, the expression of GFP and of LPA_1_ (measured by intensity of the fluorescent signal) was significantly increased in MTLE mice compared to control ones (Figures 3C,D). These results further validate the expression of GFP in LPA_1_-GFP mice as a reliable correlate of LPA_1_ expression. Although we did not detect LPA_1_-GFP^+^ cells outside the SGZ and the GCL, for example, the molecular layer where reactive gliosis takes place, we wanted to confirm that RAs did not express LPA_1_-GFP using S100β as a differential marker (Figure 3E). We injected LPA_1_-GFP mice with a single dose of KA in the cortex (cx) where in normal condition there is no expression of LPA_1_-GFP and analyzed them 3 days later. The injection of KA triggered reactive astrogliosis as visualized by immunostaining for GFAP and S100β (Supplementary Figures 3A,B). No expression of LPA_1_-GFP (Supplementary Figures 3A,B) or of LPA_1_ by immunostaining (Supplementary Figure 3C) was found in the cx of either control or KA mice. This result rules out that astrocytes could start the expression of LPA_1_-GFP when they transform into RAs after an excitotoxic insult. As positive controls for the staining, we checked the DG in the same slices for expression of GFAP, S100β, and LPA_1_-GFP (Supplementary Figures 3D,E). For positive immunostaining of LPA_1_ (see Figures 2A,B). A scheme of the combination of biomarkers that differentially identify NSCs, React-NSCs, astrocytes and RAs is provided in Supplementary Figure 2E. Astrocytes express GFAP and S100β but not nestin (or nestin-GFP) or LPA_1_-GFP. Finally, RAs express nestin (and nestin-GFP), GFAP, and S100β, but not LPA_1_-GFP.

These results strongly suggest that the expression of LPA_1_-GFP is indeed a specific marker of React-NSCs, at least for 2 weeks, but later they gradually lose the expression of LPA_1_-GFP/GFAP as they convert into RAs. As we have previously reported (Sierra et al., 2015), astrocytes do not increase their low rate of cell division in MTLE, and close to zero GFAP^+^GFP^–^ cells incorporated BrdU (data not shown). Most of the BrdU^+^ cells were indeed GFAP^+^GFP^+^, which allows us to rely on BrdU staining to trace the differentiation of LPA_1_-GFP^+^ cells. As expected, LPA_1_-GFP^+^/GFAP^+^ did not express S100β in the earlier time points (Supplementary Figure 3A). Over time, the percentage of LPA_1_-GFP^+^/GFAP^+^ cells expressing also S100β increased gradually so that most of the remaining LPA_1_-GFP^+^/GFAP^+^ cells expressed S100β after 10 weeks (Supplementary Figures 3B–D).

Another noticeable finding from the time course is that after several weeks neurons in the GCL started to express LPA_1_-GFP (Supplementary Figures 4A–C) only in the MTLE mice. Although staining was less intense than that of React-NSCs, the proportion of granule cells (colabeled with the neuronal marker NeuN) increased over time after 2 weeks so that approximately 25% of all neurons in the GCL expressed LPA_1_-GFP 10 weeks after the KA injection (Supplementary Figure 4D). Colocalization of LPA_1_-GFP with NeuN was absent in the control mice. Lysophosphatidic acid receptor 1–GFP–positive neurons were located throughout the GCL without any particular location (Supplementary Figure 4B), and we did not find colocalization of LPA_1_-GFP^+^/NeuN^+^ neurons with BrdU (data not shown). These observations suggest that preexistent mature granule cells are the ones that start to express LPA_1_ rather than newborn ones.

**FIGURE 4:**
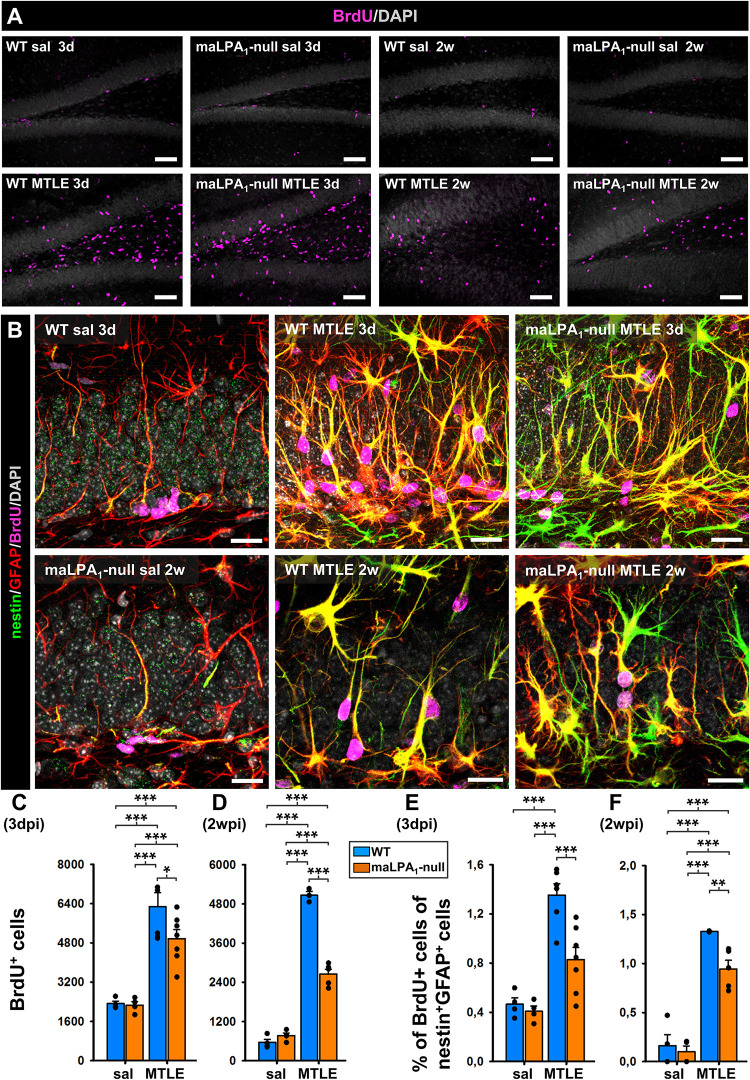
Absence of LPA_1_ reduces the massive activation of React-NSCs. WT and maLPA_1_-null mice were subjected to the model of MTLE (or the corresponding control) and then were administered BrdU (four injections, 2 h apart) on the second day after the saline/KA intrahippocampal injection. The mice were sacrificed 3 days or 2 weeks after surgery. We proceeded to quantify the total number of BrdU cells in the DG **(A)** and the proportion of dividing (BrdU^+^) NSCs and React-NSCs **(B)** by confocal microscopy. The number of BrdU cells was much higher in MTLE mice, but it was reduced in the maLPA1 compared to the WT at 3 days **(C)**. These differences were more evident at the 2-week time point **(D)**. At 3 days, the proportion of dividing (BrdU^+^) NSCs among total NSCs (identified by expression of nestin and GFAP) did not change in the control (saline) group. In MTLE WT mice the proportion of dividing NSCs was much higher than in any sal group but was reduced (without difference to sal levels) in the MTLE maLPA_1_-null mice **(E)**. 2 weeks after the induction of MTLE, the number of dividing NSCs in the MTLE maLPA_1_-null mice was also significantly reduced compared to the MTLE WT mice **(F)**, although in this case both MTLE groups were higher than the sal groups. Scale bar is 50 mm in **(A)** and 20 mm in **(B)**. Data were analyzed by a two-way ANOVA. Post hoc Holm–Sidak was used to analyze differences between WT, maLPA_1_-null, sal, and MTLE groups. *p < 0.05, **p ≤ 0.005, ***p ≤ 0.001.

Given the specific expression of LPA_1_-GFP and LPA_1_ in NSCs and in seizure-induced React-NSCs in the short term, we wondered whether LPA_1_ could play a role in the induction of React-NSCs. For this purpose, we used the maLPA_1_-null described above (Figure 1). We subjected WT and maLPA1-null mice to saline or KA intrahippocampal injection and administered BrdU during the second day after surgery (four injections, 2 h apart). Mice were then sacrificed 3 days or 2 weeks after the surgery, and slices were stained for BrdU, GFP, and GFAP (Figures 4A,B). We first quantified the number of NSCs in control WT and maLPA_1_-null mice 3 days and 2 weeks after the surgery to make sure that the basal population of NSCs was not altered because of the constitutive lack of LPA_1_ (Supplementary Figure 4E). After 3 days, cell proliferation, measured as the number of BrdU^+^ cells in the DG, did not change in the maLPA_1_-null saline-injected mice compared to the WT saline-injected ones (Figure 4A). Three days after the saline or KA injection, the total number of BrdU^+^ cells was the same in saline mice (WT and maLPA_1_-null) but was significantly increased in both MTLE groups. There was, however, a significantly lower number of dividing cells in the maLPA_1_-null MTLE mice compared to the WT MTLE mice (Figure 4C). Two weeks after the injection, the results were similar to the 3-dpi point, but the difference between WT MTLE and maLPA_1_-null MTLE mice was much larger (Figure 4D). We then quantified the proportion of dividing (BrdU^+^) NSCs (saline) or React-NSCs (MTLE mice) at 3 days or 2 weeks after saline or KA injection. At 3 dpi, there was no difference between saline WT or maLPA_1_-null mice. In the MTLE mice, the proportion of dividing React-NSCs was significantly higher than in the saline mice only in the WT mice, but not in the maLPA_1_-null animals. Accordingly, there was a smaller fraction of GFP^+^GFAP^+^BrdU^+^ in the MTLE maLPA_1_-null mice than in the MTLE WT ones (Figure 4E). At 2 weeks post-injection, results were similar to the previous time point, with MTLE maLPA_1_-null mice having a significantly reduced proportion of BrdU^+^-labeled cells than MTLE WT mice, although in this case both were higher than in any of the saline-injected mice (Figure 4F). We conclude that, indeed, the expression of LPA_1_ regulates (at least in a cell-autonomous manner) NSC division in pathological conditions and that it could be targeted to prevent massive activation of seizure-induced React-NSCs.

## Discussion

With the overall purpose of characterizing the transformation from hippocampal NSCs to React-NSCs and finally into RAs induced by seizures, we searched for new tools that selectively labeled this cell lineage. There has been a lack of specific markers for adult hippocampal NSCs, and this is one of the reasons why the characterization of their properties was elusive for many years after the discovery of adult hippocampal neurogenesis. Nestin, the protein “specifically expressed in neuroepithelial stem cells” (Lendahl et al., 1990), is the most used marker of NSCs, especially through the development of transgenic mice, which express GFP under the regulatory elements of nestin (Yamaguchi et al., 2000; Mignone et al., 2004). Nestin, however, is expressed also in the neurogenic precursors born from NSCs besides other cell types outside the neurogenic cascade such as NG2-positive oligodendrocyte progenitor cells and pericytes (Yamaguchi et al., 2000; Filippov et al., 2003; Encinas et al., 2011). Furthermore, the expression of nestin is one of the most characteristic properties of RAs (Clarke et al., 1994). Thus, the identification of NSCs, and especially of the RA-looking React-NSCs in conditions in which the generation of RAs is induced such as MTLE, can be troublesome. Recently, two new lines of transgenic mice have been characterized in which specific labeling of hippocampal NSCs has been reported. One is the *Lfng*-eGFP, in which lunatic fringe drives the expression of the enhanced GFP (Semerci et al., 2017), and the other is the LPA_1_-GFP based on the expression of LPA_1_ (Walker et al., 2016). We have herein used the LPA_1_-GFP transgenic line of mice to further validate its expression in LPA_1_-expressing cells (through anti-LPA_1_ immunostaining in WT and maLPA1 mice). We have also characterized the onset of its expression in postnatal NSCs and tested the hypothesis that it could be also a good marker of React-NSCs. Under pathological conditions such as seizures (Sierra et al., 2015; Muro-García et al., 2019) or stroke (Faiz et al., 2015) adult-brain NSCs generate RAs at the expense of their neurogenic potential. In the hippocampus, in models of MTLE and before differentiating into RAs, hippocampal NSCs transform into React-NSCs, different from both normal NSCs and RAs. React-NSCs divide in much higher rate than NSCs and overexpress nestin and GFAP as RAs do, but in contrast, they do not express S100β at least for 4 weeks after the onset of seizures (Sierra et al., 2015; Muro-García et al., 2019). We have also confirmed that reactive astrogliosis induced by an excitotoxic insult (injection of KA in the cx) does not induce the expression of LPA_1_ or LPA_1_-GFP. In other words, RAs do not express LPA_1_-GFP as we anticipated by the lack of expression of LPA_1_ or LPA_1_-GFP outside the SGZ and GCL in control and MTLE mice. We have here described how React-NSCs overexpress LPA_1_ and LPA_1_-GFP early after the induction of seizures and keep expressing LPA_1_-GFP for several weeks, whereas no other cell type does. Later, as they transform into RAs and the expression of S100β starts, they lose the expression of LPA_1_-GFP. This is an important finding because it tells us that at least for several weeks React-NSCs are different from NSCs and from RAs and that indeed we can use LPA_1_-GFP as a specific marker of React-NSCs to further explore their properties in the future. The combination of biomarkers that (together with morphology) can be used to unequivocally identify NSCs, React-NSCs, astrocytes, and RAs is summarized in Supplementary Figure 2D). Interestingly, as the expression of LPA_1_-GFP is downregulated in React-NSCs, it becomes upregulated in neurons of the GCL. Lysophosphatidic acid receptor expression could play a role in neuronal function as its activation by gintonin has been reported to enhance both excitatory and inhibitory synaptic transmission in the adult rat hippocampus (Park et al., 2015). More related to our study, LPA signaling has been proposed to play a role in the regulation of KA-induced cell death, although this was especially observed in CA3 and the receptor whose expression was affected in the DG was LPA_3_ and not LPA_1_ (Lee et al., 2014). In this same line, the analysis of mouse KA-induced epileptic seizure models has demonstrated notable changes of LPA levels in dorsal hippocampus at acute seizure phase (Lerner et al., 2018). For the moment, we can only speculate LPA_1_ expression could be part of the group of pathological hallmarks of MTLE such as GCL dispersion and that could be related to neuronal cell damage.

Regarding the role of LPA_1_ in hippocampal neurogenesis, brains of the maLPA_1_-null mice show defects in proliferation in the DG and neurogenesis (Estivill-Torrus et al., 2008; Matas-Rico et al., 2008). Furthermore, *in vivo* administration of the endogenous ligand LPA promoted neurogenesis *in vivo* (Walker et al., 2016). We have, however, not found differences in the basal population of NSCs, suggesting that the alterations would be due to changes in the mitotic capabilities of NSCs and progenitors. We have here used the maLPA_1_-null mice in our model of MTLE to address its functional role within the neurogenic niche in pathological conditions. We found that the lack of LPA_1_ indeed prevented the massive activation (cell division) of React-NSCs (measured 3 days or 2 weeks after MTLE), which translated in an overall reduced number of BrdU^+^ cells in the DG 2 weeks after the induction of MTLE. An overall effect in cell proliferation was not noticed in the earliest time point (3 days after MTLE) most likely because the impact on React-NSCs division was not enough to make an impact on overall cell division. Thus, we conclude that LPA_1_ is particularly expressed in NSCs and seizure-indeed React-NSCs and that it acts as a specific marker for React-NSCs at least for 3 to 4 weeks, before they differentiate into RAs, and neurons of the GCL start to express it. Further, we show that LPA_1_ plays a role in one of the key features of React-NSCs, their massive activation due to neuronal hyperexcitation. This finding opens the door to the possibility of targeting LPA_1_ to preserve the population of NSCs in MTLE.

## Data Availability Statement

The raw data supporting the conclusions of this article will be made available by the authors, without undue reservation.

## Ethics Statement

All procedures were approved by the University of the Basque Country (UPV/EHU) Ethics Committees (Leioa, Spain) and Diputación Foral de Bizkaia. All procedures followed the European directive 2010/63/UE and NIH guidelines.

## Author Contributions

RV-M and SM-S participated in the experimental design, performed the experiments and data analysis, prepared the figures, and co-wrote the manuscript. TM-G participated in the experimental design, performed experiments and data analysis, and prepared the figure regarding LPA_1_-GFP validation in normal and MTLE mice. OP-A participated in the experimental design, performed experiments and data analysis, and prepared the figure regarding postnatal analysis. GE-T participated in the experimental design and provided the maLPA_1_-null mice as well as the resources to work with them. GE-T and FR generated the maLPA_1_-null mice. JE designed the project, participated in the experimental design, performed experiments and data analysis, prepared the figures, provided funding, and co-wrote the manuscript. All authors contributed to the article and approved the submitted version.

## Conflict of Interest

The authors declare that the research was conducted in the absence of any commercial or financial relationships that could be construed as a potential conflict of interest.
