# Corrigendum: Lysophosphatidic Acid Receptor 1 Specifically Labels Seizure-Induced Hippocampal Reactive Neural Stem Cells and Regulates Their Division

**DOI:** 10.3389/fnins.2020.614857

**Published:** 2020-11-16

**Authors:** Roberto Valcárcel-Martín, Soraya Martín-Suárez, Teresa Muro-García, Oier Pastor-Alonso, Fernando Rodríguez de Fonseca, Guillermo Estivill-Torrús, Juan Manuel Encinas

**Affiliations:** ^1^The Neural Stem Cell and Neurogenesis Laboratory, Achucarro Basque Center for Neuroscience, Leioa, Spain; ^2^Department of Neurosciences, University of the Basque Country (UPV/EHU), Leioa, Spain; ^3^Unidad de Gestión Clínica de Salud Mental, Hospital Regional Universitario de Málaga, Instituto de Investigación Biomédica de Málaga (IBIMA), Málaga, Spain; ^4^Unidad de Gestión Clínica de Neurociencias, Hospital Regional Universitario de Málaga, Instituto de Investigación Biomédica de Málaga (IBIMA), Málaga, Spain; ^5^Ikerbasque, The Basque Foundation for Science, Bilbao, Spain

**Keywords:** neural stem cells, hippocampal neurogenesis, seizures, lysophosphatidic acid receptor 1, gliosis

An author name was incorrectly spelled as ^**^Guillermo Estivil-Torrús^**^. The correct spelling is ^**^Guillermo Estivill-Torrús^**^.

The authors apologize for this error and state that this does not change the scientific conclusions of the article in any way. The original article has been updated.

