# Supplementary material for: Lysophosphatidic Acid Receptor 1 Specifically Labels Seizure-Induced Hippocampal Reactive Neural Stem Cells and Regulates Their Division
Source: Front Neurosci. 2020 Aug 14;14:811. doi: 10.3389/fnins.2020.00811 (PMC7456947; doi:10.3389/fnins.2020.00811)
Supplement: Supplementary file 1 [file Data_Sheet_1.pdf]

Lysophosphatidic acid receptor 1 specifically labels seizure-induced hippocampal reactive neural stem cells and regulates their division.

Supplementary Materials and Methods

Animals

LPA_1-GFP transgenic mice, generated by the GENSAT project at Howard Hughes Medical Institute (The Rockefeller University, NY, USA) (Gong et al., 2003), were provided by Dr. Gerd Kempermann at the Center for Regenerative Therapies Dresden (Technische Universität Dresden, Dresden, Germany) and crossbred with C57BL/6 mice for at least 10 generations. Animals were housed with ad libitum food and water access, in 12:12h light cycle, and were 2 months old at the time of the start of the experiments. All procedures were approved by the University of the Basque Country EHU/UPV Ethics Committees (Leioa, Spain) and Diputación foral de Bizkaia under protocol M20/2015/236. All procedures followed the European directive 2010/63/UE and NIH guidelines. For the studies regarding the absence of LPA_1, mice of the spontaneous variant maLPA_1-null (Estivill-Torrus et al., 2008), derived from previously reported LPA_1-null (Contos et al., 2000); and their WT counterparts (on a mixed background C57BL/6 x 129SW) were kindly provided by Guillermo Estivill-Torrús at the Instituto de Investigación Biomédica de Málaga (IBIMA, Hospital Regional de Málaga, Málaga, Spain).

Model of MTLE (stereotaxic intrahippocampal injection of KA).

We followed the protocol optimized and described in (Sierra et al., 2015; Abiega et al., 2016; Bielefeld et al., 2017). Mice were anesthetized with intraperitoneal ketamine (Dechra Veterinary Products, Barcelona, Spain)/medetomidine (Braun VetCare, Tuttlingen, Germany) (75:1 mg/kg) and received a single dose of the analgesic buprenorphine (1mg/kg) (Animalcare, York, United Kingdom) subcutaneously. After positioning in the stereotaxic apparatus, a 0.6mm hole was drilled at coordinates taken from Bregma to target the DG (based on (Franklin and Paxinos, 1997)): anteroposterior (AP) -1.8mm, laterolateral (LL) -1.6mm. A pulled glass microcapillary was inserted at -1.9mm dorsoventral (DV), and 50nl of saline (NaCl 0.9%, Sal group) or KA (Sigma-Aldrich, St Louis, MO, USA) 20mM (1nmol, MTLE group) were delivered into the right hippocampus using a microinjector (Nanoject II, Drummond Scientific, Broomal, PA, USA). After 2min, the microcapillary was retracted, and the mice sutured and maintained on a thermal blanket until recovered from anesthesia. The animals were continuously monitored during the first hours following the procedure and once or twice daily after the first 24h. **Injection of KA in the cortex.** The procedure and the dose of sal and KA where the same as for the model of MTLE but we used the coordinates: - -0.1mm AP, 1.8mm LL, -2mm DV (right cx) for KA and -0.1mm AP, +1.8mm LL, -2mm DV (left cx) for sal.

BrdU administration

BrdU was diluted in sterile phosphate-buffered saline (PBS) with 0.01N sodium hydroxide and administered through intraperitoneal injections at 150mg/kg concentration. LPA_1-GFP and maLPA_1-null/WT mice were given four injections separated by 2-h intervals on the second day after intrahippocampal injection of KA or Sal.

Immunohistochemistry

Immunohistochemical techniques were performed essentially as described before following methods optimized for the use in transgenic mice (Encinas and Enikolopov, 2008; Encinas et al., 2011). Animals were subjected to transcardial perfusion with 30ml of PBS followed by 30ml of 4% (w/v) paraformaldehyde (PFA) in PBS, pH 7.4. The brains were removed and postfixed, with the same fixative, for 3h at room temperature, then transferred to PBS and kept at 4ºC. Due to antigen sensitivity, mice whose brain sections were destined to LPA_1 immunostaining were perfused with periodate lysine PFA (PLP) fixative consistent of 0.01M sodium metaperiodate, 0.075M lysine and 4% PFA in 0.1M phosphate buffer (PB) (McLean and Nakane, 1974). The same fixative was used for overnight postfixation.

Serial 50µm-thick sagittal sections were cut using a Leica VT 1200S vibratome (Leica Microsystems GmbH, Wetzlar, Germany). Immunostaining was carried out following a standard procedure: the sections were incubated with blocking and permeabilization solution containing 0.25% Triton-X100 and 3% bovine serum albumin (BSA) in PBS for 3h at room temperature, and then incubated overnight with the primary antibodies (diluted in the same solution) at 4ºC. After thorough washing with PBS, the sections were incubated with fluorochrome-conjugated secondary antibodies diluted in the permeabilization and blocking solution for 3h at room temperature. After washing with PBS, the sections were mounted on slides with Dako fluorescent mounting medium (Dako, Carpinteria, CA). Those sections destined to the analysis of BrdU incorporation were treated, before the immunostaining procedure, with 2M HCl for 20min at 37ºC, rinsed with PBS, incubated with 0.1M sodium tetraborate for 10 min at room temperature, and then rinsed with PBS. The GFP signal from the transgenic mice was detected with an antibody against GFP for enhancement and better visualization.

The following primary antibodies were used: chicken anti-GFP (Aves laboratories, GFP-1020, 1:1,000), chicken anti-Nestin (Aves laboratories, NES, 1:1,000), rat anti-BrdU (Bio-Rad, MCA2060, 1:400), rabbit anti-GFAP (Dako, Z0334, 1:1,500), goat anti-GFAP (Abcam, Ab53554, 1:1,000), rabbit anti-NeuN (Abcam, Ab177487, 1:500), rabbit anti-S100 (Dako, Z0311, 1:500), and rabbit anti-LPA_1 (EDG2, ThermoFisher Scientific, PA1-1041, 1:100). The secondary antibodies used, all in 1:500 concentration, were: goat anti-chicken Alexa Fluor 488 (ThermoFisher Scientific, A11039), donkey anti-chicken FITC (Rockland, 603-702-C37), donkey anti-rabbit Alexa Fluor 488 (ThermoFisher Scientific, A21206), donkey anti-rabbit Alexa Fluor 568 (ThermoFisher Scientific, A10043), donkey anti-rat Alexa Fluor 594 (ThermoFisher Scientific, A21209), donkey anti-rabbit Alexa Fluor 680 (ThermoFisher Scientific, A10043), and donkey anti-goat Alexa Fluor 680 (ThermoFisher Scientific, A21084). 4′,6-diamidino-2-phenylindole (DAPI, Sigma-Aldrich), at 1:1,000, was also added to the sections during the incubation with the secondary antibodies to counterstain cell nuclei.

Image capture

All fluorescence immunostaining images were collected employing a Leica SP8 (Leica, Wetzlar, Germany) laser scanning microscope and LAS X software. Images were exported as tiffs and adjusted for brightness, contrast, and background, equally for the entire image, using the “levels” tools in Adobe Photoshop without any further modification. All images shown are flat projections, or orthogonal projections, from z-stacks of approximately 10µm of thickness.

Cell quantification

Quantitative analysis of cell populations in constitutive transgenic mice was performed by design-based (assumption free, unbiased) stereology using a modified optical fractionator-sampling scheme as previously described (Encinas and Enikolopov, 2008; Encinas et al., 2011). Hemispheres were sliced sagittally in a lateral-to-medial direction including the entire dentate gyrus (DG). Slices were collected using systematic-random sampling. The 50-µm slices were collected in 6 parallel sets, each set consisting of approximately 12 slices, each slice 250 µm apart from the next.

Cells were categorized following the criteria described previously (Encinas and Enikolopov, 2008; Encinas et al., 2011). NSCs were defined as radial glia-like LPA_1-GFP^+GFAP^+ or $nestin^+GFAP^+$ cells with the cell body located in the subgranular zone (SGZ) or the lower third of the granule cell layer (GCL) and with an apical process extending from the SGZ towards the molecular layer (ML) through the GCL. At the 2w-time point of the $maLPA_1$-null/WT mice experiments, though, we did not take into account the radial morphology due to the expected morphological change induced by seizures, but quantification was restricted to the SGZ and the GCL and considering that normal, parenchymal astrocytes divide very rarely in both control and MTLE mice. Neurons were defined as $NeuN^+$ cells with round big nucleus located in the GCL. All absolute quantifications were performed with the 40x magnification, capturing frames of known volume (typically 100w x 100h x 10d). Density was then normalized for the total surface of the SGZ of the DG, measured at 40x. The volume of the DG was not used (the height of the SGZ plus the GCL was excluded) because the GCL changes significantly with MTLE due to cell dispersion.

For proportional quantifications (with BrdU), representative numbers ranging from 50 to 300 cells of interest were quantified and classified in randomly selected confocal z-stacks from 30µm below to 30µm above the GCL using a 63x oil immersion objective. For total numbers of BrdU-positive cells, all $BrdU^+$-nuclei in the SGZ and the GCL were counted following bias-free stereology employing a Zeiss ApoTome2 (Carl Zeiss, Jena, Germany) microscope with a 40x oil immersion objective.

To measure the expression of LPA_1-GFP and LAP_1 by their fluorescence signal after antibody staining, LPA_1-GFP^+ and LAP_1^+ cells were manually selected in z-stacks using the “Threshold” tool to outline only the pixels of the image with each respective staining. Then using the “Measure” tool we calculated the integrated density, which is the sum of all the pixel intensities (in arbitrary units). A minimum of 30 cells from five hippocampal sections from 3 different mice for each condition (sal and MTLE) were analyzed.

Statistical analysis

SigmaPlot (San Jose, CA, USA) was used for statistical analysis. A one-way ANOVA was performed to analyze data for the postnatal expression of LPA_1-GFP by NSCs (Supplementary Figure 1) and for the expression of LPA_1-GFP by $NeuN^+$ neurons (Supplementary Figure 2). When evaluating the time x treatment interaction in the LPA_1-GFP time course a two-way ANOVA was employed, and analyses continued when normality assumptions were fulfilled. Otherwise data were converted to logarithmic scale (Figure 6D). For post-hoc analysis of data a Holm-Sidak test was used. When variances were not homogeneous (by Levene´s test) a Mann-Whitney U (Wilcoxon rank sum) test was used instead (Figure 3C,D). Only $p<0.05$ is reported to be significant. Data are shown as mean ± standard error of the mean (SEM).

Supplementary Figures.

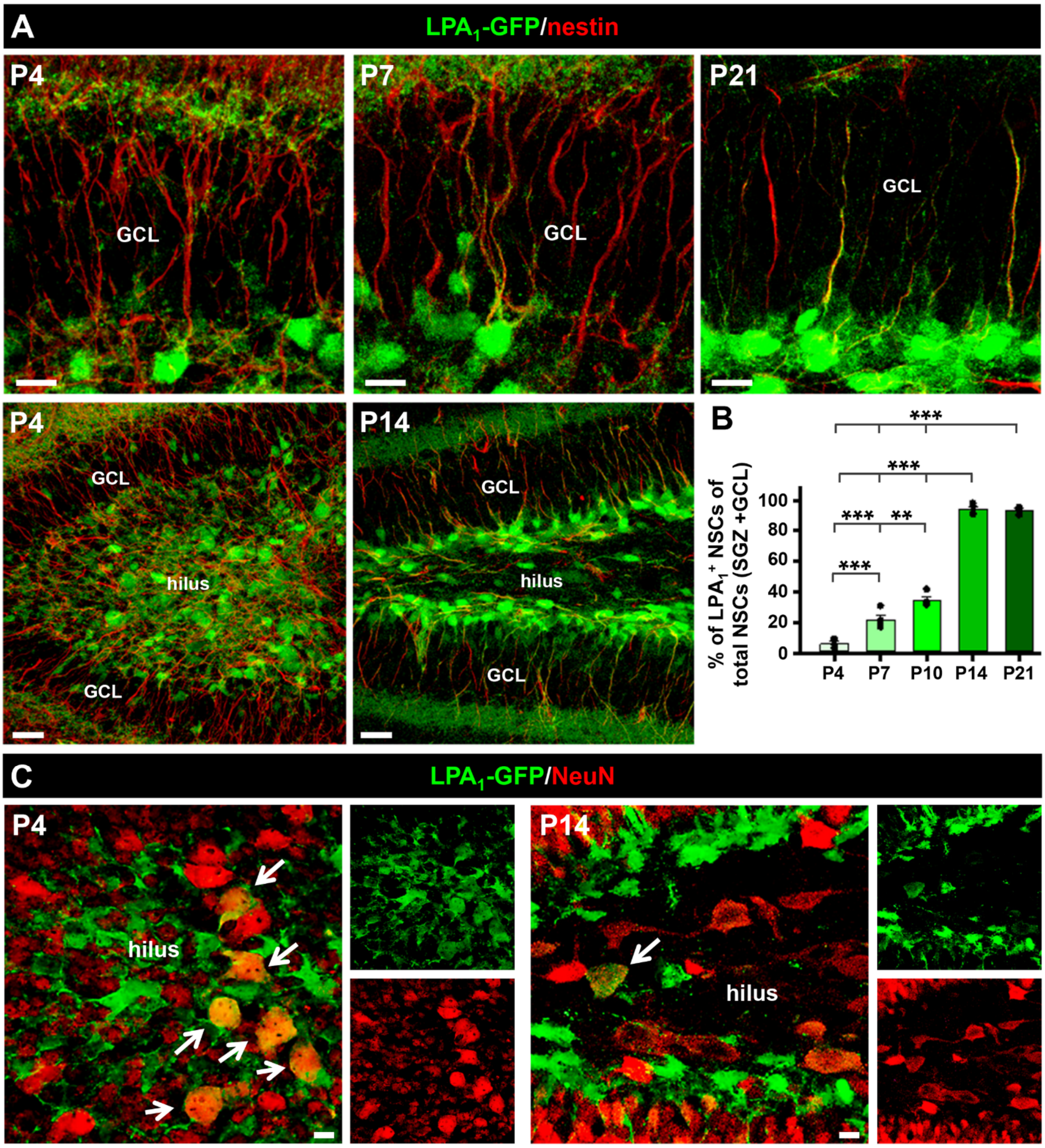

SUPPLEMENTARY FIGURE 1. NSCs start to express LPA_1-GFP early postnatally. Confocal microscopy image analysis performed in LPA_1-GFP mice at 4, 7, 14 and 21 postnatal days **(A)** showed that nestin$^+$ radial cells located in the SGZ and the GCL only started to express LPA_1-GFP at P4 in very low proportion and then expression increased progressively so that at P14 and P21 all the NSCs were positive for LPA_1-GFP **(B)**. At early postnatal days a large population of NeuN$^+$ neurons in the hilus expressed LPA_1-GFP. Expression of LPA_1-GFP decreased, however, over time and was almost absent by P14 **(C)**. Scale bar is 10 µm in **(A)** and **(C)**. Data were analyzed by a one-way ANOVA that showed a statistically significant effect of time ($p<0.001$) Post hoc Holm-Sidak was used to analyze differences between time points. Graphs show individual data, mean and SEM. **$p\leq0.005$; ***$p\leq0.001$.

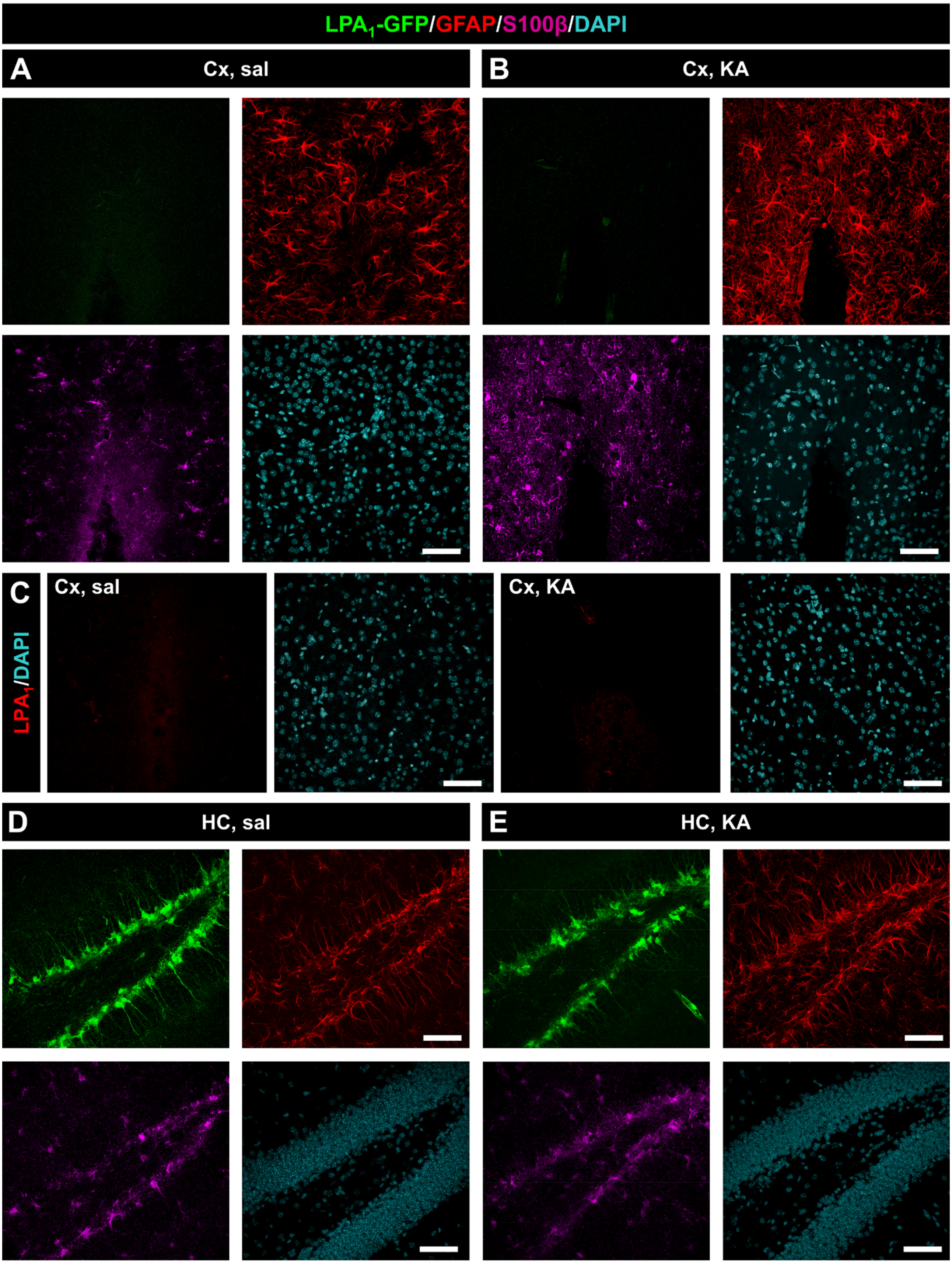

SUPPLEMENTARY FIGURE 2. LPA_1-GFP mice were injected in the cx with saline **(A)** or with KA **(B)** (and sacrificed after 3 days) to elicit an excitotoxic reactive gliosis. The confocal microscopy images show staining for LPA1-GFP, GFAP, S100β and DAPI. The staining for anti-LPA_1 antibody was also negative **(C)**. Only residual signal was found for LPA_1-GFP. In the same slices used for **(A)** and **(B)** and with the same microscopy setting, strong LPA1-GFP signal was found in the hippocampal NSCs (**D**). Scale bar is 50 µm in **(A)**, **(B)**, **(C)** and **(D)**.

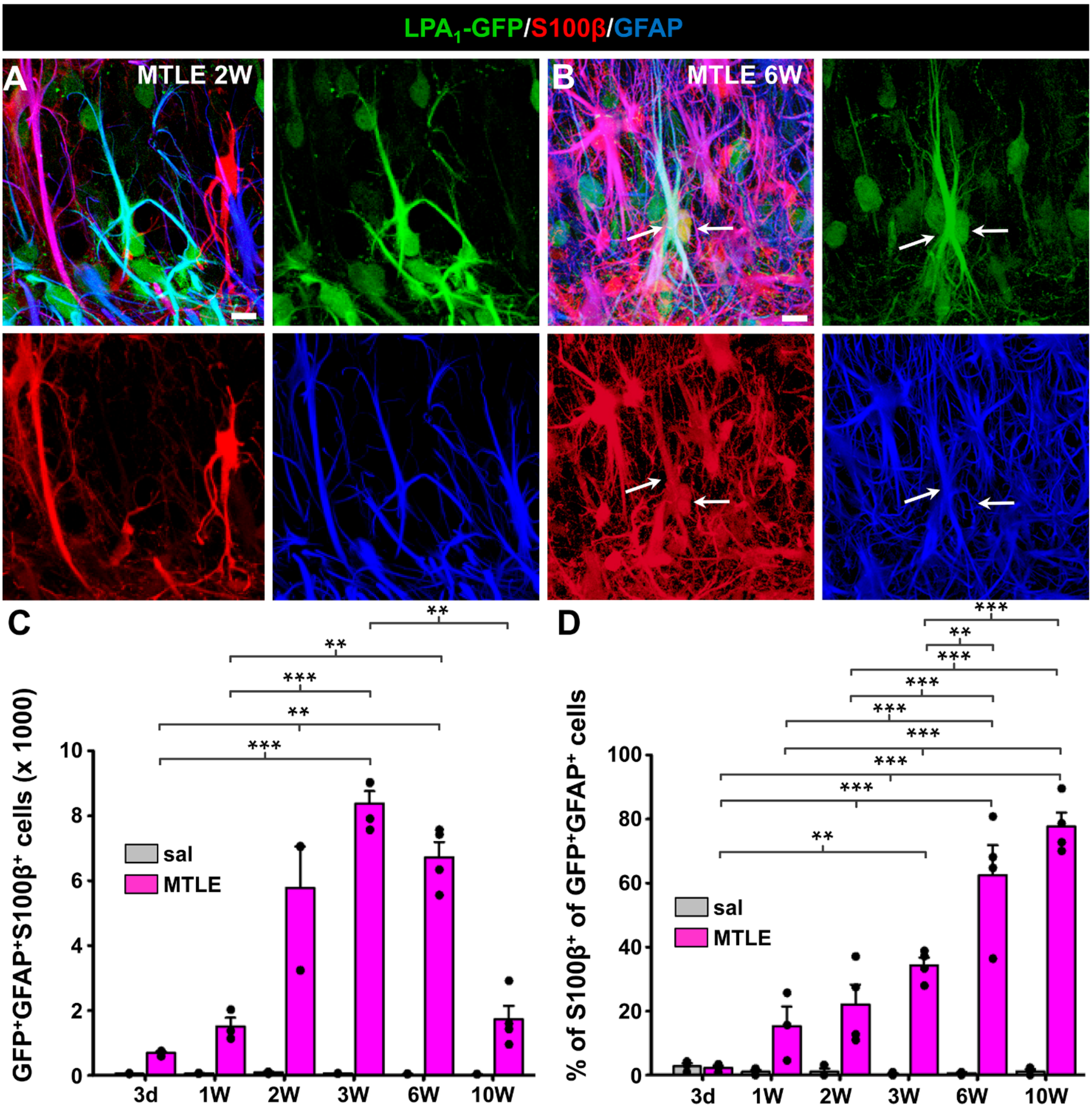

SUPPLEMENTARY FIGURE 3. Seizure-induced React-NSCs express LPA_1-GFP but lose its expression as they convert into RAs. The colocalization of LPA_1-GFP and the marker of astrocytes and RAs S100β (which is absent in NSCs) and GFAP was analyzed over time by confocal microscopy in control **(A)** and MTLE mice **(B)**. Arrows point at a LPA_1-$GFP^+GFAP^+S100\beta^+$ cell. The number of LPA_1-$GFP^+GFAP^+S100\beta^+$ cells in MTLE mice was higher than in control mice in all the time points and increased progressively over time, peaking at the 3-week time point to decrease by 10 weeks **(C)**. The percentage of $S100\beta^+$ cells among LPA_1-GFP^+GFAP^+ cells started to be higher in MTLE mice after 1 week and then progressively increased, being maximal at the last time point of analysis (10 weeks). Scale bar is 10 µm in **(A)** and **(B)**. Data were analyzed by a two-way ANOVA. A significant effect of time on the MTLE group was found in both cases **(C)** and **(D).** Post hoc Holm-Sidak was used to analyze differences between time points. Graphs show individual data, mean and SEM. **$p \leq 0.005$; ***$p \leq 0.001$.

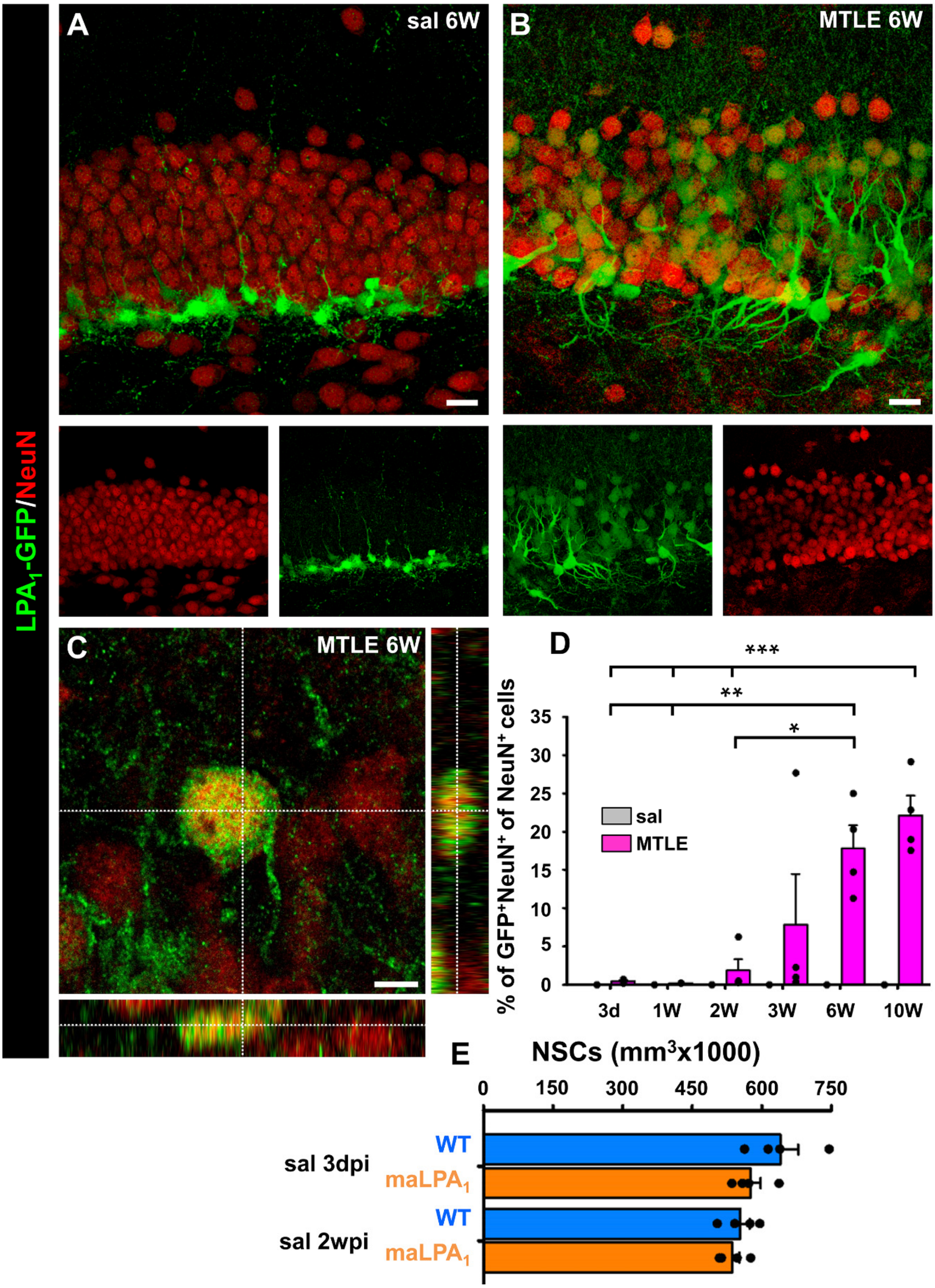

SUPPLEMENTARY FIGURE 4. Granule cells start to express LPA_1-GFP^+ weeks after the induction of MTLE. No neurons were found expressing LPA_1-GFP^+ in the control mice at any time point as analyzed by confocal imaging **(A)**. Expression in neurons of the GCL was however observed in MTLE mice **(B)** and **(C)**. The quantification shows that neurons in the GCL started to express LPA_1-GFP^+, although in a very low percentage, two weeks after the induction of MTLE. The expression of LPA_1-GFP^+ then increased progressively reaching almost 25% after 10 weeks **(D)**. **(E)** The density of NSCs in the WT and $maLPA_1$ does not change in control (saline-injected) mice 3 days or 1 week after the surgery. Scale bar is 10 µm. Data were analyzed by a one-way ANOVA as data for saline mice was zero in all the time points. Post hoc Holm-Sidak was used to analyze differences between time points in MTLE. *$p<0.05$; **$p\leq0.005$; ***$p\leq0.001$.

Supplementary References.

Abiega, O., Beccari, S., Diaz-Aparicio, I., Nadjar, A., Laye, S., Leyrolle, Q., et al. (2016). Neuronal Hyperactivity Disturbs ATP Microgradients, Impairs Microglial Motility, and Reduces Phagocytic Receptor Expression Triggering Apoptosis/Microglial Phagocytosis Uncoupling. *PLoS Biol* 14(5)**,** e1002466. doi: 10.1371/journal.pbio.1002466.

Bielefeld, P., Mooney, C., Henshall, D.C., and Fitzsimons, C.P. (2017). miRNA-Mediated Regulation of Adult Hippocampal Neurogenesis; Implications for Epilepsy. *Brain Plast* 3(1)**,** 43-59. doi: 10.3233/BPL-160036.

Contos, J.J., Fukushima, N., Weiner, J.A., Kaushal, D., and Chun, J. (2000). Requirement for the lpA1 lysophosphatidic acid receptor gene in normal suckling behavior. *Proc Natl Acad Sci U S A* 97(24)**,** 13384-13389. doi: 10.1073/pnas.97.24.13384.

Encinas, J.M., and Enikolopov, G. (2008). Identifying and quantitating neural stem and progenitor cells in the adult brain. *Methods Cell Biol* 85**,** 243-272. doi: 10.1016/S0091-679X(08)85011-X.

Encinas, J.M., Michurina, T.V., Peunova, N., Park, J.H., Tordo, J., Peterson, D.A., et al. (2011). Division-coupled astrocytic differentiation and age-related depletion of neural stem cells in the adult hippocampus. *Cell Stem Cell* 8(5)**,** 566-579. doi: 10.1016/j.stem.2011.03.010.

Estivill-Torrus, G., Llebrez-Zayas, P., Matas-Rico, E., Santin, L., Pedraza, C., De Diego, I., et al. (2008). Absence of LPA1 signaling results in defective cortical development. *Cereb Cortex* 18(4)**,** 938-950. doi: 10.1093/cercor/bhm132.

Franklin, K.B.J., and Paxinos, G. (1997). *The Mouse Brain in Stereotaxic Coordinates*. San Diego: Academic Press.

Gong, S., Zheng, C., Doughty, M.L., Losos, K., Didkovsky, N., Schambra, U.B., et al. (2003). A gene expression atlas of the central nervous system based on bacterial artificial chromosomes. *Nature* 425(6961)**,** 917-925. doi: 10.1038/nature02033.

McLean, I.W., and Nakane, P.K. (1974). Periodate-lysine-paraformaldehyde fixative. A new fixation for immunoelectron microscopy. *J Histochem Cytochem* 22(12)**,** 1077-1083. doi: 10.1177/22.12.1077.

Sierra, A., Martin-Suarez, S., Valcarcel-Martin, R., Pascual-Brazo, J., Aelvoet, S.A., Abiega, O., et al. (2015). Neuronal hyperactivity accelerates depletion of neural stem cells and impairs hippocampal neurogenesis. *Cell Stem Cell* 16(5)**,** 488-503. doi: 10.1016/j.stem.2015.04.003.
